# Supplementary material for: SLITRK5 is a negative regulator of hedgehog signaling in osteoblasts
Source: Nat Commun. 2021 Jul 29;12:4611. doi: 10.1038/s41467-021-24819-w (PMC8322311; doi:10.1038/s41467-021-24819-w)
Supplement: Supplementary file 1 — Supplementary Information [file 41467_2021_24819_MOESM1_ESM.pdf]

# Supplementary Information

## SLITRK5 is a negative regulator of hedgehog signaling in osteoblasts

Jun Sun[1†], Dong Yeon Shin[1, 2†], Mark Eiseman[1], Alisha R Yallowitz[1], Na Li[3], Sarfaraz Lalani[1], Zan Li[1], Michelle Cung[1], Seoyeon Bok[1], Shawon Debnath[1], Sofia Jenia Marquez[1], Tommy E. White[4], Abdul G Khan[4], Ivo C Lorenz[4], Jae-Hyuck Shim[5], Francis S. Lee[6], Ren Xu[3]*, Matthew B. Greenblatt[1, 7]*

[1] Department of Pathology and Laboratory Medicine Weill Cornell Medicine, New York, NY, USA

[2] Current Address: Research Center, LegoChem BioSciences, INC., Daejeon 34302, South Korea

[3]State Key Laboratory of Cellular Stress Biology, School of Medicine, Xiamen University, Xiamen, Fujian361102, China

[4]Tri-Institutional Therapeutics Discovery Institute, New York 10021, United States

[5]Division of Rheumatology, Department of Medicine, University of Massachusetts Medical School, 364 Plantation Street, Worcester, MA01605, USA

[6]Department of Psychiatry, Weill Cornell Medical College, New York, NY 10065, USA

[7] Research Division, Hospital for Special Surgery, New York, NY 10021, USA

† These authors contributed equally to this work.

*Correspondence:

Ren Xu

86-592- 2880577

xuren526@xmu.edu.cn

Matthew B. Greenblatt

1-212-746-1602

mag3003@med.cornell.edu

## Supplementary Figure 1

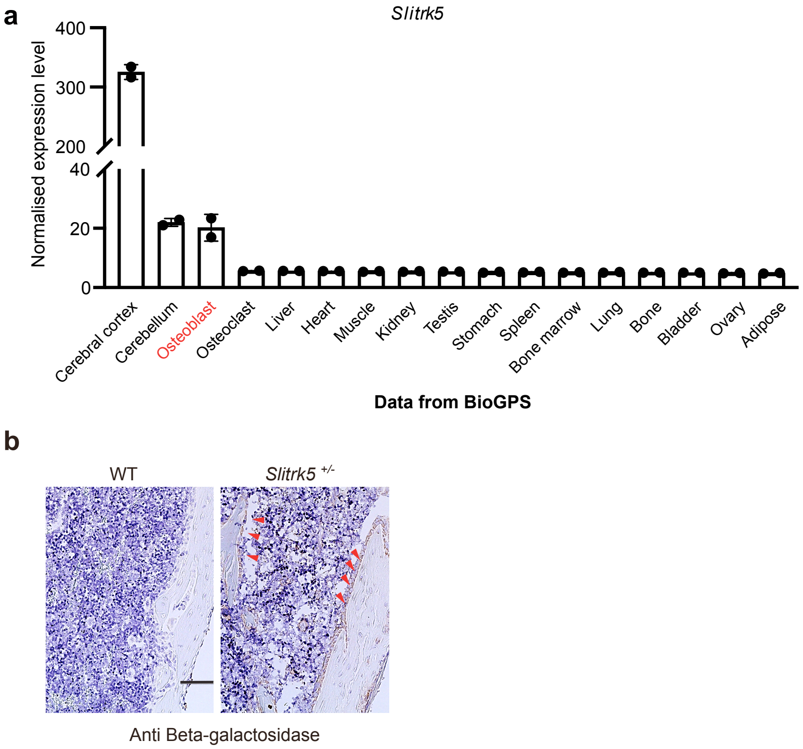


**Supplementary Figure 1. a** Slitrk5 mRNA level in different cells and tissues. The raw expression data were obtained from BioGPS (http://biogps.org/). Data are presented as mean±s.d., n=2 biologically independent samples. **b** Immunostaining of mouse femur sections with beta-galactosidase antibody, indicating the expression of Slitrk5 in osteoblasts.Data are representative of two independent experiments, scale bar=100 um.

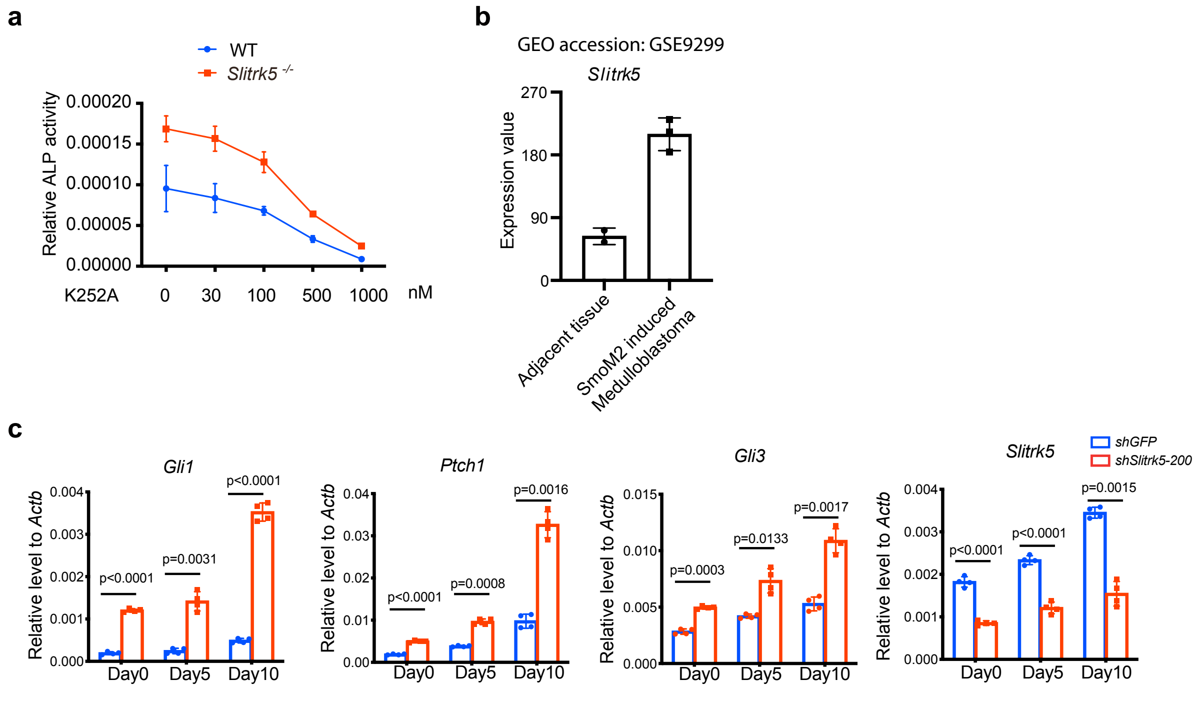


**Supplementary Figure 2. a** Primary osteoblasts from WT and Slitrk5-/- mice were treated with the indicated doses of K252a and cultured in osteoblast differentiation medium. ALP activity was measured at day 8 of osteoblast differentiation. Data are presented as mean±s.d. n=10 biologically independent samples. **b** Slitrk5 mRNA level in SmoM2 induced medulloblastoma and adjacent tissue (GEO: GSE9299). Data are presented as mean±s.d., n=3 biologically independent samples. **c** Human osteoblast-like cells (Saos-2) infected with Slitrk5 or GFP shRNA and cultured in osteoblast differentiation medium. mRNA level of Slitrk5, Gli1, Ptch1 and Gli3 at 0, 5 and 10 days of differentiation was assessed by RT-PCR. Data are presented as mean±s.d., n=4 biologically independent samples, 2-tailed unpaired t-test.

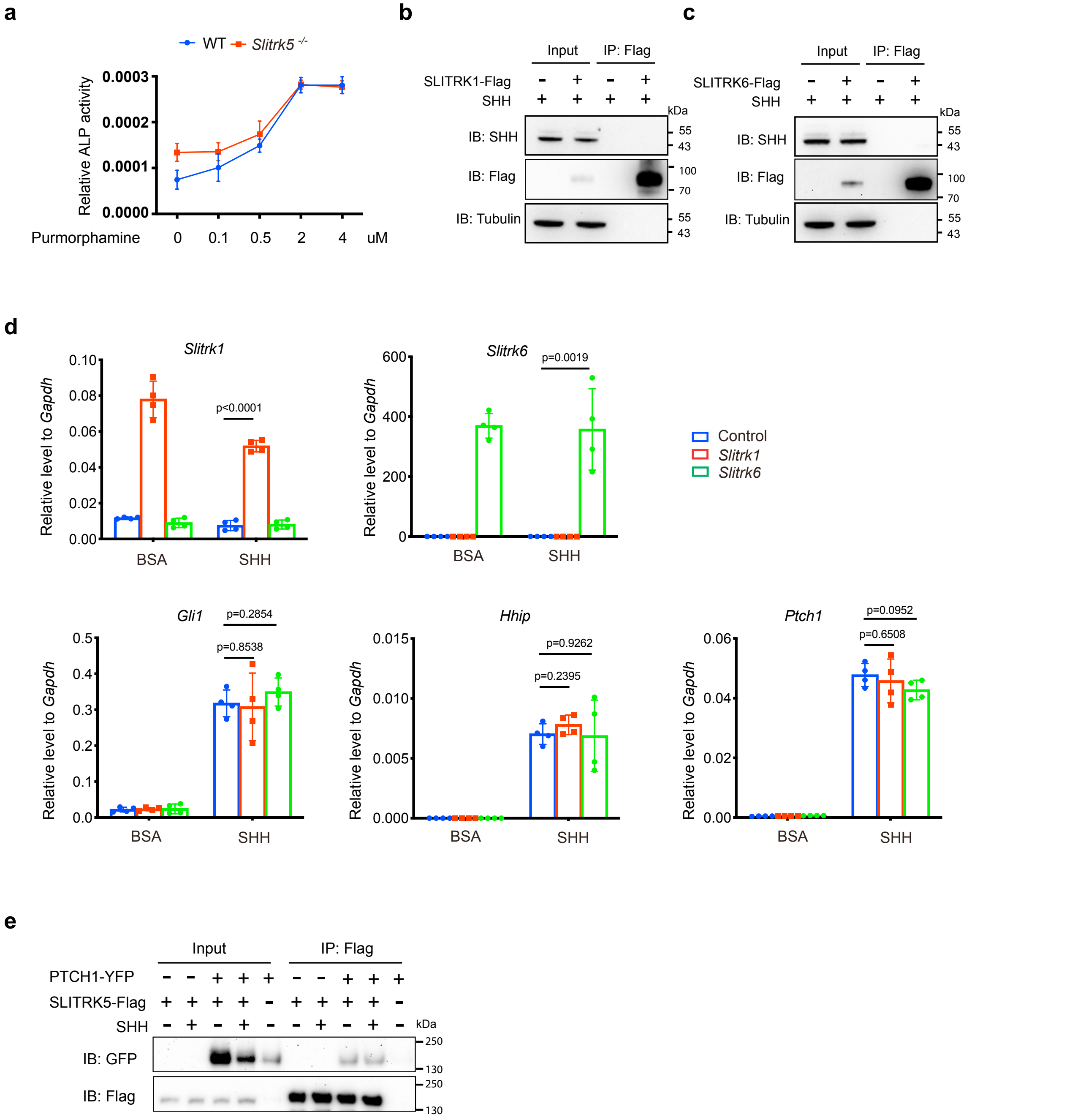


**Supplementary Figure 3. a** Primary osteoblasts from WT and Slitrk5-/- mice were treated with the indicated doses of Purmorphamine and cultured in osteoblast differentiation medium. ALP activity was measured at day 8 of differentiation. Data are presented as mean±s.d. n=10 biologically independent samples. **b** Co-immunoprecipitation of Flag-SLITRK1 and SHH in HEK293T cells. Data are representative of two independent experiments. **c** Co-immunoprecipitation of Flag-SLITRK6 and SHH in HEK293T cells. Data are representative of two independent experiments. **d** C3H10T1/2 cells transfected with either control, Slitrk1 or Slitrk6 overexpression vectors were treated with BSA or 100ng/ml SHH in serum free medium for 48h. Gli1, Ptch1, Hhip, Slitrk1 and Slitrk6 mRNA levels were measured by RT-PCR. n=4 biologically independent samples, 2-tailed unpaired t-test. **e** Co-immunoprecipitation of Flag-SLITRK5 and PTCH1-YFP in C3H10T1/2 cells. Data are representative of two independent experiments.

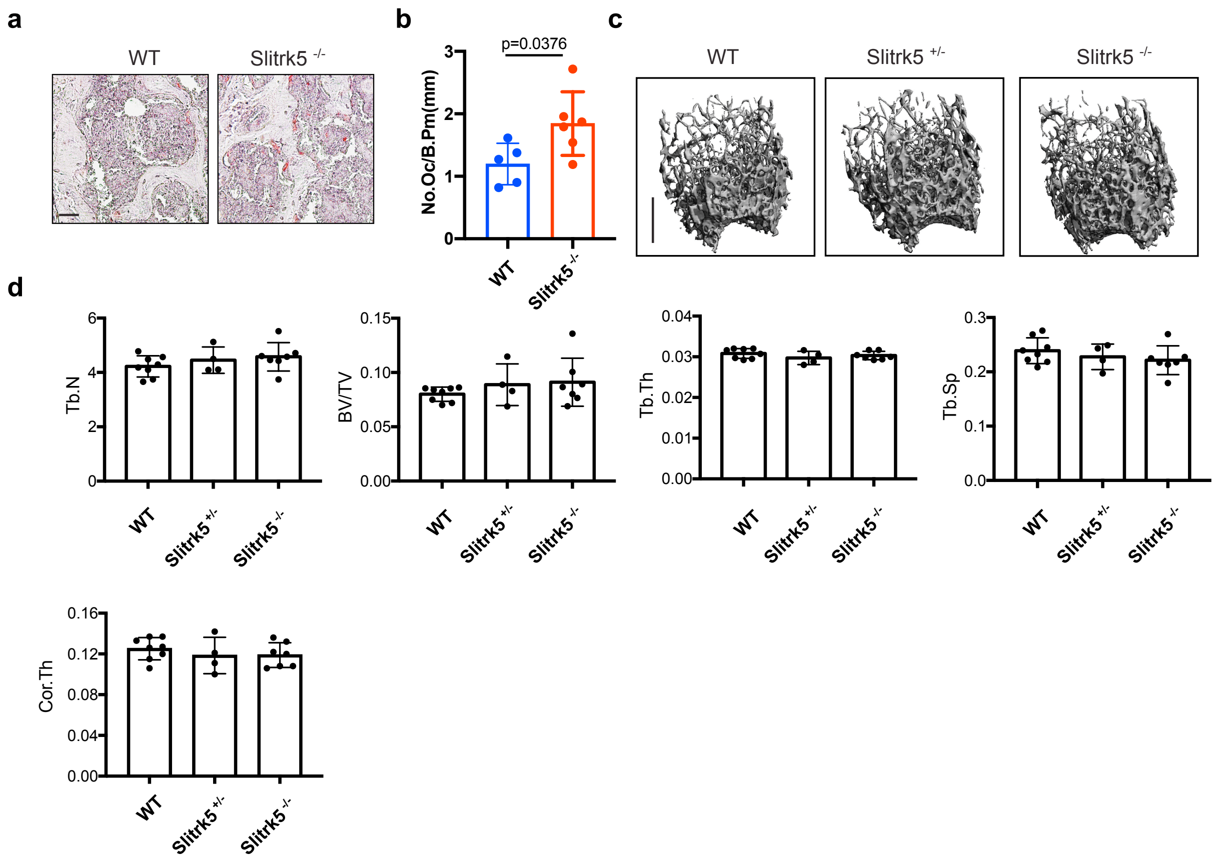


**Supplementary Figure 4. a&b** Representative images of TRAP staining (a) and quantification of No.Oc/B.Pm (b) of the L3 vertebrae in 7-week-old WT and Slitrk5-/- female mice. osteoclast number/bone perimeter (No.Oc./B.Pm) . N=5 or 6 per group. Data are presented as mean±s.d, 2-tailed unpaired t-test, scale bar=100um. **c&d** Representative images of uCT (c) and quantification of bone volume/total volume (BV/TV), trabecular number per cubic millimeter (Tb.N), trabecular thickness (Tb.Th) , trabecular separation (Tb.Sp) and cortical bone thickness (Cor.Th)(d) of femurs in WT, Slitrk5+/- and Slitrk5-/- female mice at 7-week-old. N=4-7 per group. Data are presented as mean±s.d, 2-tailed unpaired t-test, scale bar=1mm.

## Supplementary Figure 5

**Figure 3b**

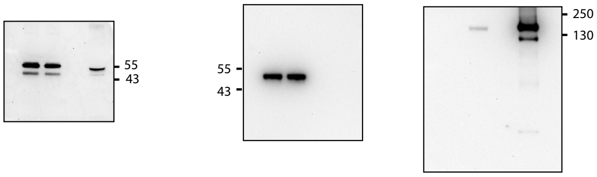


**Figure 3c**

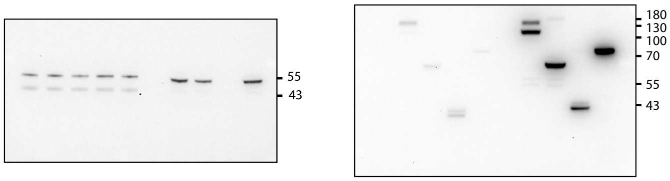


**Figure 3f**

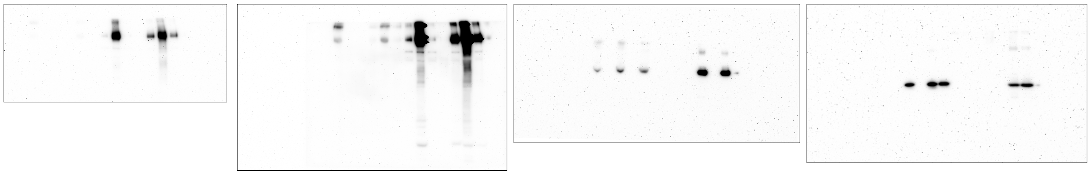

**Figure 3g**

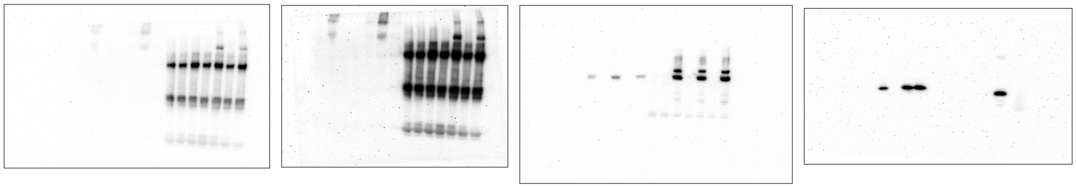

**Figure 3h**

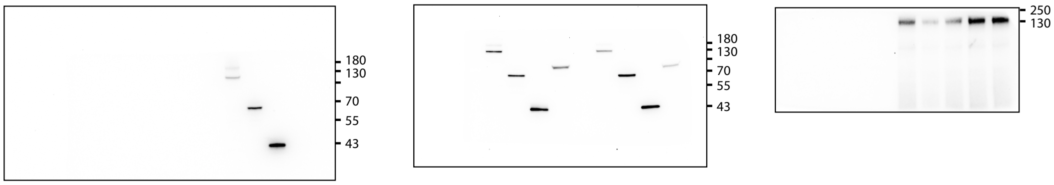


**Supplementary Figure 3b**

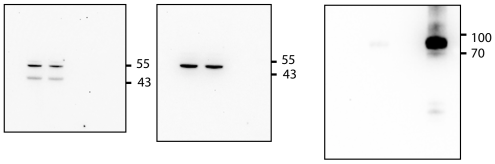


**Supplementary Figure 3c**

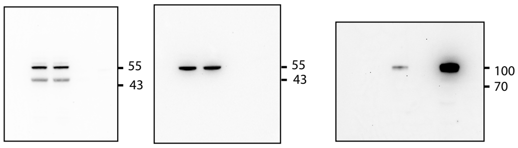


**Supplementary Figure 3e**

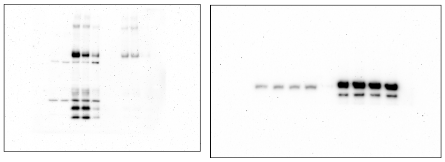

## Supplementary Table

| Name | Species | shRNA primers |
|---|---|---|
| shSlitrk5-199 | Mouse | CCGGCCTCCTGCACCTGGGTAATAACTCGAGTTATTACCCAGGTGCAGGAGGTTTTTG |
| shSlitrk5-200 | Mouse | CCGGTGCAGAAACCATCGATTATTACTCGAGTAATAATCGATGGTTTCTGCATTTTTG |
| sh-control | Mouse | CCGGGCGCGATAGCGCTAATAATTTCTCGAGAAATTATTAGCGCTATCGCGCTTTTT |
| | | |
| | | **qPCR primers** |
| Slitrk5-forward | Mouse | CGCAGAACAGACTTCCTGGAAG |
| Slitrk5-reverse | Mouse | TCAGCCTCTCAATCCTGTTGCC |
| Slitrk6-forward | Mouse | AACAACCCACCATACAACGGA |
| Slitrk6-reverse | Mouse | TTCCTCAACTTCTTCCAAATGCT |
| Bsg-forward | Mouse | AGAGGACACAGGCACTTACGAG |
| Bsg-reverse | Mouse | GACTTCCTGGACAGAGGTTTGG |
| Hhip-forward | Mouse | GAGAGATCCTGTGTGGTGGCTT |
| Hhip-reverse | Mouse | GCTGCATTCTGAGTTGTTGGTG |
| Hprt-forward | Mouse | TCAGTCAACGGGGGACATAAA |
| Hprt-reverse | Mouse | GGGGCTGTACTGCTTAACCAG |
| Gli1-forward | Mouse | CCAAGCCAACTTTATGTCAGGG |
| Gli1-reverse | Mouse | AGCCCGCTTCTTTGTTAATTTGA |
| Ptch1-forward | Mouse | AAAGAACTGCGGCAAGTTTTTG |
| Ptch1-reverse | Mouse | CTTCTCCTATCTTCTGACGGGT |
| Ptch2-forward | Mouse | GGCACTCACATCCGTCAACAAC |
| Ptch2-reverse | Mouse | GAAGACGAGCATTACCGCTGCA |
| Ocn-forward | Mouse | TGCTTGTGACGAGGTATCAG |
| Ocn-reverse | Mouse | GTGACATCCATACTTGCAGG |
| Alp-forward | Mouse | CGGGACTGGTACTCGGATAA |
| Alp-reverse | Mouse | ATTCCACGTCGGTTCTGTTC |
| Sp7-forward | Mouse | TCACTTGCCTGCTCTGTTCC |
| Sp7-reverse | Mouse | GCGGCTGATTGGCTTCTTCT |
| Runx2-forward | Mouse | CCAACCGAGTCATTTAAGGCT |
| Runx2-reverse | Mouse | GCTCACGTCGCTCATCTTG |
| Gli2-forward | Mouse | ACACTGTGGAGGACTGCCTACA |
| Gli2-reverse | Mouse | GGCATCTCCATGCCACTGTCAT |
| Col1a1-forward | Mouse | CCTCAGGGTATTGCTGGACAAC |
| Col1a1-reverse | Mouse | CAGAAGGACCTTGTTTGCCAGG |
| Gli3-forward | Human | TCAGCAAGTGGCTCCTATGGTC |
| Gli3-reverse | Human | GCTCTGTTGTCGGCTTAGGATC |
| Gli1-forward | Human | AGCCTTCAGCAATGCCAGTGAC |
| Gli1-reverse | Human | GTCAGGACCATGCACTGTCTTG |
| Ptch1-forward | Human | GCTGCACTACTTCAGAGACTGG |
| Ptch1-reverse | Human | CACCAGGAGTTTGTAGGCAAGG |
| Slitrk5-forward | Human | CCTCCAGTACAATCTCATCCGC |
| Slitrk5-reverse | Human | TCAAGCCAGAGAAGACGCCTGA |
