# Supplementary material for: SLITRK5 is a negative regulator of hedgehog signaling in osteoblasts
Source: Nat Commun. 2021 Jul 29;12:4611. doi: 10.1038/s41467-021-24819-w (PMC8322311; doi:10.1038/s41467-021-24819-w)
Supplement: Supplementary file 2 — Reporting Summary [file 41467_2021_24819_MOESM2_ESM.pdf]

nature research

Corresponding author(s): Matthew Blake Greenblatt, Ren Xu

Last updated by author(s): Jun 14, 2021

Reporting Summary

Nature Research wishes to improve the reproducibility of the work that we publish. This form provides structure for consistency and transparency in reporting. For further information on Nature Research policies, see our Editorial Policies and the Editorial Policy Checklist.

Statistics

For all statistical analyses, confirm that the following items are present in the figure legend, table legend, main text, or Methods section.

n/a	Confirmed	
☐	☒	The exact sample size (n) for each experimental group/condition, given as a discrete number and unit of measurement
☐	☒	A statement on whether measurements were taken from distinct samples or whether the same sample was measured repeatedly
☐	☒	The statistical test(s) used AND whether they are one- or two-sided<br>*Only common tests should be described solely by name; describe more complex techniques in the Methods section.*
☒	☐	A description of all covariates tested
☐	☒	A description of any assumptions or corrections, such as tests of normality and adjustment for multiple comparisons
☐	☒	A full description of the statistical parameters including central tendency (e.g. means) or other basic estimates (e.g. regression coefficient) AND variation (e.g. standard deviation) or associated estimates of uncertainty (e.g. confidence intervals)
☐	☒	For null hypothesis testing, the test statistic (e.g. F, t, r) with confidence intervals, effect sizes, degrees of freedom and P value noted<br>*Give P values as exact values whenever suitable.*
☒	☐	For Bayesian analysis, information on the choice of priors and Markov chain Monte Carlo settings
☒	☐	For hierarchical and complex designs, identification of the appropriate level for tests and full reporting of outcomes
☒	☐	Estimates of effect sizes (e.g. Cohen's d, Pearson's r), indicating how they were calculated

Our web collection on statistics for biologists contains articles on many of the points above.

Software and code

Policy information about availability of computer code

Data collection	SCANCO Medical microCT 35 system (v4.05, Switzerland) was used for scanning and 3D analysis.<br>Carl Zeiss Zen 2.3 SP1 FP3 (black, v14.0.18.201, Germany) was used for immunofluorescence imaging analysis.<br>QuantStudio 6 Flex RT-PCR Software v1.3 was used for mRNA analysis.
Data analysis	GraphPad PRISM software (v8.4.3, La Jolla, CA) was used for statistical analysis.<br>SCANCO Medical microCT 35 system (v4.05, Switzerland) was used for scanning and 3D analysis.<br>Carl Zeiss Zen 2.3 SP1 FP3 (black, v14.0.18.201, Germany) was used for immunofluorescence imaging analysis.<br>QuantStudio 6 Flex RT-PCR Software v1.3 was used for mRNA analysis.<br>CiliaQ ImageJ plugins(CiliaQ-0.1.4,CiliaQ Editor_JNH-0.1.0 and CiliaQ Preparator_JNH-0.1.0) were used for image segmentation and ciliary fluorescence quantification

For manuscripts utilizing custom algorithms or software that are central to the research but not yet described in published literature, software must be made available to editors and reviewers. We strongly encourage code deposition in a community repository (e.g. GitHub). See the Nature Research guidelines for submitting code & software for further information.

Data

Policy information about availability of data

All manuscripts must include a data availability statement. This statement should provide the following information, where applicable:

- Accession codes, unique identifiers, or web links for publicly available datasets
- A list of figures that have associated raw data
- A description of any restrictions on data availability

All relevant data are available from the authors. Uncropped blots are provided in Supplementary Figure 5. Source data are provided with this paper. The source

data underlying Figures 1a,1d,1e,2a-2f, 3a, 4b, 4e, 4f, 5a,5d,5f,5h,5j,5m, Supplementary Figure 1a,2a,2b,2c, 3a, 3d,4b,and 4d are provided as a Source Data file. The dataset(GSE9299) used in Supplementary Figure 2b is available at https://www.ncbi.nlm.nih.gov/geo/query/acc.cgi?acc=GSE9299.

Field-specific reporting

Please select the one below that is the best fit for your research. If you are not sure, read the appropriate sections before making your selection.

☒ Life sciences ☐ Behavioural & social sciences ☐ Ecological, evolutionary & environmental sciences

For a reference copy of the document with all sections, see nature.com/documents/nr-reporting-summary-flat.pdf

Life sciences study design

All studies must disclose on these points even when the disclosure is negative.

Sample size	Generally, sample sizes were calculated on the assumption that a 30% difference in the parameters measured would be considered biologically significant with an estimate of sigma of 10-20% of the expected mean. Alpha and Beta were set to the standard values of .05 and 0.2, respectively.
Data exclusions	No data are excluded.
Replication	All experiments reported in the manuscript were replicated successfully to confirm reproducibility.<br>1) Immunoblotting on samples included at least 2 independent experiments, each with consistent results.<br>2) Animal studies were reproduced across at least 3 independent cohorts.<br>3) RT-PCR on mouse bone included at least 11 independent samples per each genotype and experimental group, respectively.<br>4) Cilia immunostaing staining studies were underwent 3 independent replicates and data from at least 40 cells were used for quantification.<br>5) Immunostaining and RNA in situ on mouse samples included at least 2 independent experiments.
Randomization	Specimens were assigned to group based on genotype. For all other studies using cell-lines, no randomization was needed and proper plating controls were included for consistency. No other randomization processes were used for other analyses.
Blinding	The investigators were blinded to group allocation during data collection and analysis.

Reporting for specific materials, systems and methods

We require information from authors about some types of materials, experimental systems and methods used in many studies. Here, indicate whether each material, system or method listed is relevant to your study. If you are not sure if a list item applies to your research, read the appropriate section before selecting a response.

Materials & experimental systems

n/a	Involved in the study	
☐	☒	Antibodies
☐	☒	Eukaryotic cell lines
☒	☐	Palaeontology and archaeology
☐	☒	Animals and other organisms
☒	☐	Human research participants
☒	☐	Clinical data
☒	☐	Dual use research of concern

Methods

n/a	Involved in the study	
☒	☐	ChIP-seq
☒	☐	Flow cytometry
☒	☐	MRI-based neuroimaging

Antibodies

Antibodies used	Primary antibodies used for immunoblotting and immunofluorescence staining were specific for anti-acetylated tubulin (Sigma, T7451, clone 6-11b-1,0000106162,1:1000), anti-tubulin (Santa Cruz, sc-23948,H160,C2316,1:5000), anti-HA (cell signaling technology,3724, C29F4, 12/2017,1:2000),anti-DYKDDDDK (cell signaling technology,14793s, D6W5B, 11/2018,1:2000), anti-GFP (Abcam, ab13970, GR89472-25,1:5000),anti-β-galactosidase antibody (GTX77365; GeneTex,1:100), anti-SHH (H-160, sc-9024, Santa Cruz,1:1000), anti-SHH (E1, sc-365112, Santa Cruz,1:1000), anti-Osteopontin (AF808-SP, R&D systems1:400), and anti-Flag (9696S, Cell Signaling Technology, 1:1000).
Validation	The following validation method was conducted for IF and IHC using anti-acetylated tubulin (Sigma, T7451, clone 6-11b-1,0000106162,1:1000),anti-β-galactosidase antibody (GTX77365; GeneTex,1:100),anti-DYKDDDDK (cell signaling technology,14793s, D6W5B, 11/2018,1:2000) or anti-Osteopontin (AF808-SP, R&D systems1:400): secondary antibody was added alone without primary mouse antibody addition as negative control. The validation statements of the other antibodies can be found on the manufacturer's website.

Eukaryotic cell lines

Policy information about cell lines

Cell line source(s)	All three cell lines used in the paper were purchase from ATCC : C3H10T1/2 cells clone 8 (CCL-226), 293T (ATCC® CRL-3216™),Saos-2 (ATCC® HTB-85™).
Authentication	None of the cell lines used were authenticated.
Mycoplasma contamination	Cells were tested for mycoplasma using the Plasmo Test-Mycoplasma Detection Kit from Invitrogen and PCR analysis. All cells were confirmed to be free of mycoplasma.
Commonly misidentified lines (See ICLAC register)	No commonly misidentified cell lines were used in the study.

Animals and other organisms

Policy information about studies involving animals; ARRIVE guidelines recommended for reporting animal research

Laboratory animals	Slitrk5-/- mice were imported from Dr.Francis S. Lee's lab at Weill Cornell Medicine. Both female and male mice were used for the study. 7-week-old mice were used for uCT and histomorphometry analysis. 6-week-old mice were used for femur bone fracture model. 5-7 days old mice were used for primary calvarial osteoblasts isolation and 3-week-old mice were used for bone marrow stromal cells isolation. Mice were maintained in a controlled environment with 12:12 light-dark cycle, room temperature of 20.5–22.5 °C and humidity of 30-70%, and had ad libitum access to dry laboratory food and water.
Wild animals	No wild animals were used.
Field-collected samples	This study did not involve field-collected samples.
Ethics oversight	All experiments were performed according to the guidelines approved by the Institutional Animal Care and Use Committee of Weill Cornell Medical College.

Note that full information on the approval of the study protocol must also be provided in the manuscript.
